# Why are archaea not pathogenic? A hypothesis based on metabolism-habitat covariation

**DOI:** 10.1128/mbio.01185-26

**Published:** 2026-08-21

**Authors:** Daniel J. Harrison, Matthew S. Fullmer, Nobuto Takeuchi

**Affiliations:** 1 School of Biological Sciences, University of Auckland 99026, Auckland, New Zealand; 2 Universal Biology Institute, University of Tokyo 594897, Tokyo, Japan; 3 Department of Biology, Faculty of Sciences, Kyushu University 98328, Fukuoka, Japan; National Institutes of Health, Bethesda, Maryland, USA

**Keywords:** archaea, pathogenicity, microbial ecology, microbial metabolism, chemotrophy, organotrophy, heterotrophy, extremophile

## Abstract

Pathogenicity, the ability to cause infectious diseases in multicellular eukaryotes, is widespread among bacteria and eukaryotes but has not been reliably identified in archaea. To explore possible reasons for this disparity, we perform comparative analyses of thousands of bacterial and archaeal isolates. Our results show that isolated bacterial pathogens possess the ability to use organic compounds as sources of energy, electrons, and carbon (chemo-organo-heterotrophy or COH), suggesting that COH is a metabolic prerequisite for pathogenicity. Moreover, we find that isolated archaea capable of COH do not inhabit multicellular eukaryotes and instead predominantly inhabit extreme environments that preclude such eukaryotes. In contrast, all isolated archaea that inhabit multicellular eukaryotes are incapable of COH metabolism. This metabolism-habitat covariation in isolated archaea, together with COH as a potential prerequisite for pathogenicity in bacteria, suggests that the absence of archaeal pathogens may be due to a combination of the two factors: COH-capable archaea lack the environmental opportunity to inhabit multicellular eukaryotes, while non-COH archaea lack the metabolic prerequisite for pathogenicity.

**IMPORTANCE:**

Pathogenicity—the ability to cause infectious disease—is widespread among bacteria and eukaryotes but conspicuously absent from archaea, the third domain of life. To understand why archaea are non-pathogenic, we performed comparative analyses of thousands of bacterial and archaeal isolates. We found that the absence of pathogenic archaea can be explained by a combination of metabolic and environmental factors. Specifically, archaea living within eukaryotic, multicellular hosts lack a metabolic capability correlated with pathogenicity—chemo-organo-heterotrophy—whereas those possessing this metabolism typically live under extreme conditions, such as high temperature, and therefore lack the environmental opportunity to interact with eukaryotic hosts. These findings advance our understanding of microbes by revealing important links between their metabolism, habitats, and pathogenicity.

## INTRODUCTION

Pathogenicity, the ability to infect and cause disease in multicellular eukaryotes, is widespread across Bacteria and Eukarya. Of approximately 54 bacterial phyla (1), 13 contain known pathogens (2). Similarly, among 55 eukaryotic phyla, 18 contain known pathogens (1). In striking contrast, Archaea consist of 39 phyla (1, 3, 4), but no pathogenic archaeon has ever been reliably identified (5–11). This contrasting situation is also notable when the frequency of pathogens is considered at the species level: if the frequency of human pathogens in archaeal species were the same as that in bacteria, there would be roughly 54 species of archaeal human pathogens (2, 6). Why do archaea appear to be non-pathogenic to eukaryotic, multicellular hosts?

Some authors argue that pathogenic archaea exist but remain unobserved owing to the underdevelopment of detection methods (3, 6, 11). They suggest that archaea, especially methanogens, are, in principle, capable of pathogenicity since they possess several essential traits for pathogenicity, such as the capability to colonize the host, the ability to compete or coexist with members of the host microbiota, the ability to evade the host immune system, and the possession of putative virulence factors including secretion pathways and toxin production (3, 6, 11, 12). Motivated by this argument, extensive efforts have been made to identify pathogenic methanogens, resulting in the detection of at least nine archaeal species in diseased tissue (13). However, none of these species is considered pathogenic, as they do not satisfy Koch’s postulates (11) or other more recently developed criteria to establish microbial causation of disease (14–16). Instead, current data favor the notion that these archaea metabolically reshape the local environment, supporting the growth of bacterial pathogens, rather than infecting and subsequently directly harming their hosts (11, 12, 17, 18). Thus, despite these extensive research efforts, to date, no archaeal pathogen has been conclusively identified (11, 19). While this conspicuous absence of pathogenic archaea does not rule out their existence, it suggests that they are at best rare relative to bacterial pathogens, warranting investigation into what factors might contribute to this rarity.

One such factor, proposed by Gill and Brinkman (2011), is that Archaea lack access to virulence factor genes (VFGs), the genes essential for pathogenesis (9). They argue that Archaea cannot acquire VFGs because they are not infected by bacteriophages and therefore lack access to bacterial gene pools, a key reservoir of VFGs. However, this hypothesis is challenged by genomic studies indicating that horizontal gene transfer from Bacteria to Archaea can be mediated by alternative mechanisms, such as plasmid conjugation (20–24), which can also disseminate VFGs (25, 26). In fact, the gut archaeon *Methanobrevibacter smithii* has been reported to have acquired putative bacterial VFGs through plasmid-mediated horizontal transfer (27). Given that archaea can acquire VFGs through mechanisms other than bacteriophages, their limited access to bacteriophage gene pools does not appear to be a sufficient explanation for the rarity of archaeal pathogens.

Other authors hypothesize that archaea are non-pathogenic because of their inherent metabolic constraints, which we term metabolic constraint hypotheses (7, 10, 28). To our knowledge, three such hypotheses have been put forward. The first hypothesis, proposed by Martin, posits that pathogens must be able to use the same cofactors (e.g., vitamins) as those used by their hosts (28). Under this hypothesis, archaea are non-pathogenic, as they use distinct, archaeal-specific cofactors (28). The second hypothesis, proposed by Valentine, posits that pathogens must be able to thrive in environments involving highly fluctuating availability of energy sources, such as host-derived organic compounds, during infection (7). This hypothesis further posits that archaea lack this ability as they are low-energy specialists, which prefer stable environments and possess traits limiting their adaptability, such as a narrow niche range and reliance on highly specialized metabolic pathways for energy acquisition (7). The third hypothesis, proposed by Abedon, posits that pathogens must be able to metabolize “complex” organic compounds, such as glucose (see Discussion) (10). Under this hypothesis, archaea are non-pathogenic because this ability is rarely possessed by archaea living under non-extreme conditions (10).

While these metabolic constraint hypotheses emphasize different aspects of archaeal metabolism, they share one assumption, namely, that archaea are less capable of metabolically exploiting organic compounds that are abundantly present in multicellular eukaryotes. This prompted us to identify the core metabolic abilities of bacterial pathogens to exploit organic compounds and then to examine whether the distribution of these abilities among the archaea whose metabolic abilities are experimentally characterized sheds light on the possible absence of archaeal pathogens. Our results identify chemo-organo-heterotrophy (COH) as one such core metabolic ability and reveal that COH is widespread among archaea isolated from extreme environments but is notably absent from archaea isolated from eukaryotic hosts. Although this environment-metabolism covariation is detected in isolated archaea, if this pattern generally holds, it can constrain the evolution of pathogenicity among archaea, warranting the examination of this pattern beyond the isolated archaea.

## RESULTS

### Chemo-organo-heterotrophy as a metabolic prerequisite for bacterial pathogenicity

To determine how pathogenicity is associated with abilities to metabolize organic compounds, we compiled information about the metabolic abilities and pathogenic status of 1,891 bacterial species from IMG/M (29) and literature, as described in Materials and Methods (“Data set I: metabolic and pathogenic annotations of bacterial isolates”). We distinguished three metabolic abilities: COH, which is characterized by the ability to use organic compounds as the sources of energy, electrons, and carbons; non-COH, which is characterized by the ability to use inorganic compounds as one or more of these three sources (e.g., photo-organo-autotrophy); and mixotrophy, which is characterized by the ability to perform both COH and non-COH (e.g., chemo-organo-heterotrophy and photo-organo-heterotrophy). For each metabolic ability, we counted its occurrence in pathogenic and non-pathogenic bacterial species. These counts, displayed in Fig. 3, show that all pathogenic species possess COH ability, either exclusively or as part of mixotrophy. This result led us to hypothesize that COH is a metabolic prerequisite for pathogenicity.

**Fig 1:**
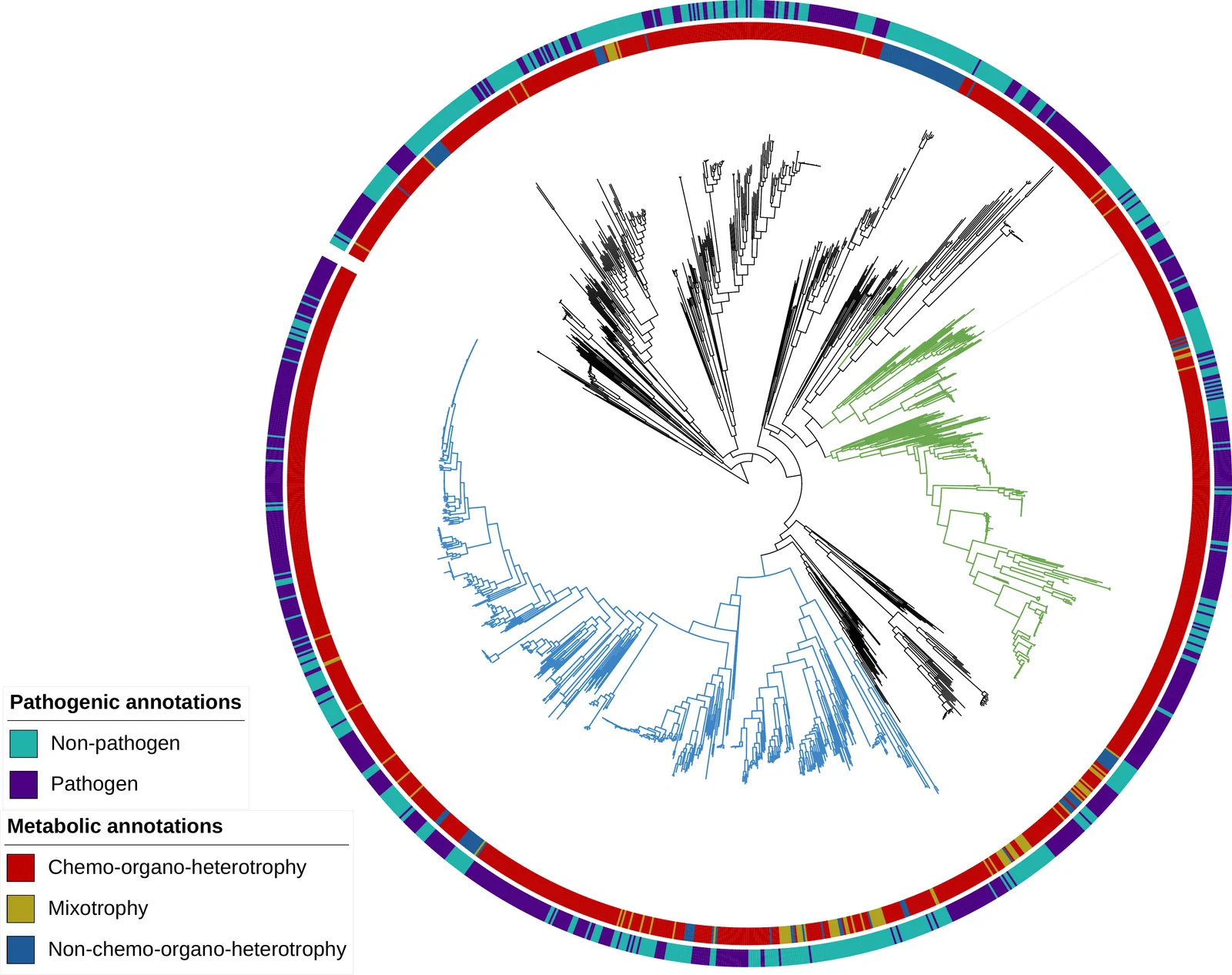
A bacterial phylogeny containing 1,462 isolates (tips) annotated with both metabolism (inner ring) and pathogen status (outer ring). The phylogeny was extracted from the MicrobesOnline database (30) and annotated using iTOL (31) as described in Materials and Methods (“Data set II: annotations of the bacterial phylogeny“). The colors of the inner ring represent the following metabolic annotations: chemo-organo-heterotrophy (red), mixotrophy (olive), and non-chemo-organo-heterotrophy (blue). The colors of the outer ring represent the following pathogenicity annotations: pathogen (purple) and non-pathogen (cyan). The colored branches correspond to Pseudomonadota (blue) and Bacillota (green) lineages. Isolates assigned an unknown metabolic or pathogenic annotation (*n* = 58) were pruned from the tree.

To examine if the association between pathogenicity and COH capability merely arises from shared evolutionary history between bacteria, we mapped the pathogenic and metabolic annotations on a bacterial phylogeny obtained from the MicrobesOnline database (30) using iTOL (31) (see Materials and Methods, “Data set II: annotations of the bacterial phylogeny”). The result shows that pathogenic bacterial isolates are scattered across different COH-capable clades (Fig. 1), suggesting that shared ancestry is not the only reason for the association between metabolism and pathogenicity.

**Fig 2:**
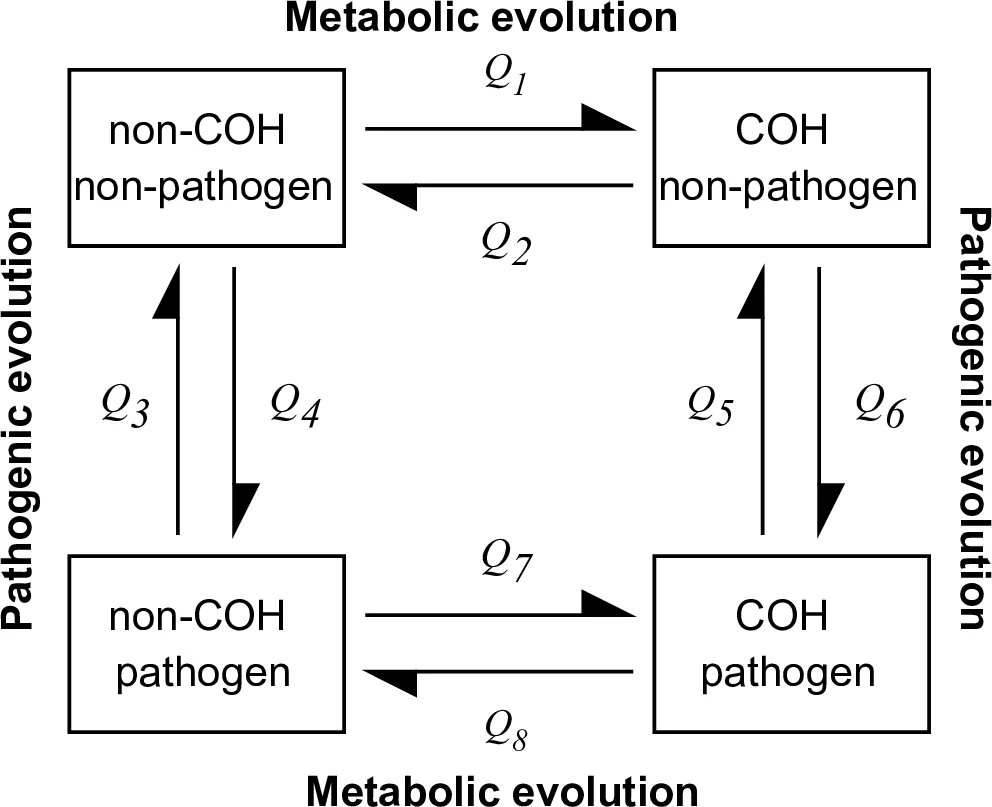
A schematic diagram of the four-state Markovian model with eight evolutionary rate parameters (model A). The arrows represent the gain or loss of a pathogen or COH metabolism. The evolutionary rate parameters are indicated above the arrows (denoted by *Q*_1_, *Q*_2_, *Q*_3_, etc.). To simplify the model, we neglected the distinction between mixotrophy and COH and regarded mixotrophy as COH. This simplification means that COH was defined as the capacity for COH in this model. The structure of this four-state Markovian model is also used in model D (see “Statistical analysis of metabolism and habitat”), with pathogen states replaced by extremophile and non-extremophile habitat preference states.

To statistically test the evolutionary association between metabolism and pathogenicity stated above, we created a Markovian model in which the evolution of pathogenicity is potentially correlated with that of metabolism (model A; Fig. 2) and compared it with a nested model in which pathogenic and metabolic evolution are uncorrelated (model B), as described in Materials and Methods (“Statistical analysis of metabolism and pathogenicity evolution”). Briefly, model A assumes four states distinguishing either pathogenic or non-pathogenic and either COH or non-COH and involves eight parameters representing transition rates between these states. Model B assumes that the transition rates between pathogenic and non-pathogenic states are independent of metabolic states, and vice versa. Fitting these models to the phylogeny in Data set II, we found that model A significantly outperforms model B (Table 1). This result indicates that the evolution of pathogenicity and that of metabolism are correlated.

**TABLE 1:** The model performance results for models A, B, and C^*b*^

Model^*a*^	lnL	AIC	AIC wt^*c*^	χ^2*d*^	*P*-value^*d*^
Model A	−753.17	1,522.34	2.1%		
	(−249.47)	(514.94)	(0%)		
Model B	−780.24	1,568.49	0%	54.15	4.9 *×* 10*^−^*^11^
	(−258.04)	(524.08)	(0%)	(17.14)	(1.8 *×* 10*^−^*^3^)
Model C	−753.32	1,514.63	97.9%	0.29	0.99
	(−253.08)	(514.16)	(100%)	(7.22)	(0.13)

^*a*^ Models B and C are nested models of model A (see Materials and Methods, “Statistical analysis of metabolism and pathogenicity evolution”).

^*b*^ The upper values correspond to model statistics of the “state-defined phylogeny,” whereas values in the parentheses correspond to model statistics of the “clade-pruned phylogeny,” which excludes isolates belonging to the Pseudomonadota and Bacillota phyla.

^*c*^ AIC weights estimate the relative plausibility of each model.

^*d*^ Test statistics (*χ*^2^) and *P*-values from likelihood-ratio tests comparing model A to nested models.

**Fig 3:**
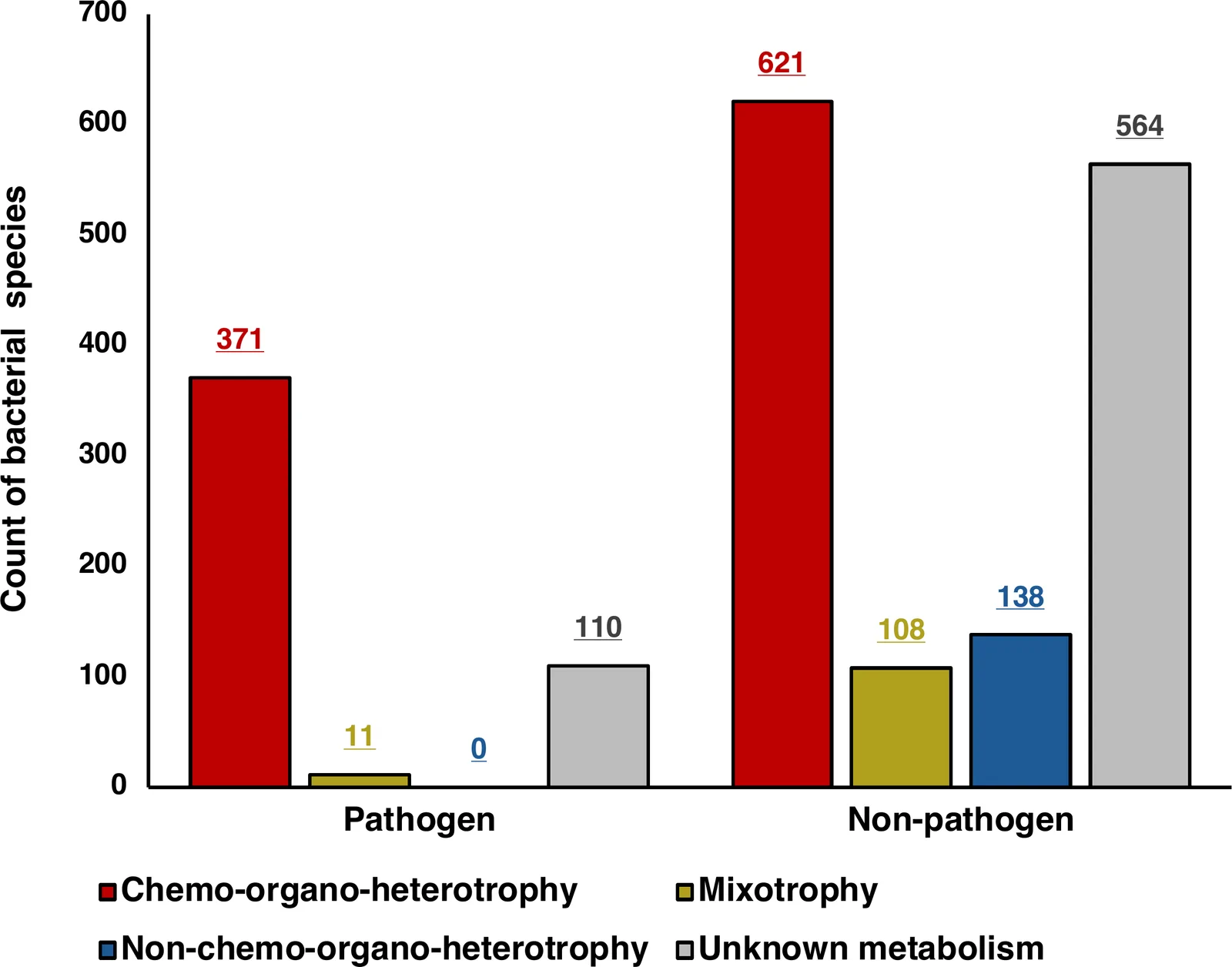
The distribution of bacterial metabolic categories among pathogenic and non-pathogenic species. This figure includes 1,923 bacterial species from the IMG/M and MicrobesOnline databases (30). Bar colors indicate the following metabolic annotations: chemo-organo-heterotroph (red), mixotroph (gold), non-chemo-organo-heterotroph (blue), and unknown (gray). Annotations were assigned using database metadata and manual literature review, as described in Materials and Methods (see “Data set I: metabolic and pathogenic annotations of bacterial isolates” and “Data set II: annotations of the bacterial phylogeny”). Species assigned an “unknown” annotation lack sufficient information to determine their metabolic category.

The absence of non-COH pathogens in our annotated bacterial data set (Data set I; Fig. 3) suggests that the ability to perform COH must evolve before the evolution of pathogenicity. To test this hypothesis, we compared model A to a model (model C) which assumes that COH capability is a prerequisite for the evolution of pathogenicity—specifically, model C lacks the non-COH pathogenic state and thus has two fewer parameters than those of model A (see Materials and Methods, “Statistical analysis of metabolism and pathogenicity evolution”). The result shows that both models have similar log-likelihood values (likelihood-ratio test *P*-value = 0.99), but the Akaike information criterion (AIC) value of model A exceeds that of model C by eight (Table 1). This suggests the data do not support the additional parameters in model A, specifically, the transitions to and from a non-COH pathogenic state. Instead, the data support model C, in which pathogenicity can only evolve from a COH state, supporting the notion that the evolution of pathogenicity must be preceded by the evolution of COH metabolism.

However, these results may be biased if particular clades are over-represented in our data, as the results would reflect the evolution of specific clades rather than across the entire phylogeny. To determine whether any bacterial phyla were over-represented in our data, we counted the number of isolates in each phylum (for further details, see “Data set II: annotations of the bacterial phylogeny” in Materials and Methods) and report these counts in the supplemental materials (Table S2). We identified two over-represented phyla: the Pseudomonadota and Bacillota.

Given the over-representation of these two phyla in our data, we tested whether our results were robust to this over-representation by repeating the statistical analyses on a tree that excludes these two phyla as described in Materials and Methods (see “Data set II: annotations of the bacterial phylogeny”). The results show that model C is better supported than models A and B based on AIC, with a similar log-likelihood to that of model A, consistent with the results mentioned above (Table 1). This indicates the results are robust to the over-representation of Pseudomonadota and Bacillota. Together, these results suggest that COH is a metabolic prerequisite for pathogenicity.

### Combination of metabolism and habitat can explain the absence of pathogenicity in isolated archaea

The above results, suggesting COH as a metabolic prerequisite for pathogenicity, led us to ask whether the lack of this prerequisite could explain the absence of pathogenicity in the isolated archaea. To investigate this question, we extracted 837 isolated archaeal species from the NCBI taxonomy database and conducted manual literature reviews to annotate them with their metabolic capabilities (see Materials and Methods, “Data set III: habitat and metabolic annotations of archaea”). Of the 837 species, 694 were annotated with either COH, non-COH, or mixotrophy (the counts of each metabolic capability are provided in the supplemental materials; see Table S1); 112 were isolated, but their metabolism was not characterized under experimental conditions and were thus assigned an unknown metabolic capability; and the remaining 31 had no available literature and could not be annotated. Importantly, we found that a great majority of the archaeal species in our data set (about 68% of 806 species) are capable of COH metabolism, indicating that the absence of COH metabolism cannot explain the absence of pathogenic archaea.

**Fig 4:**
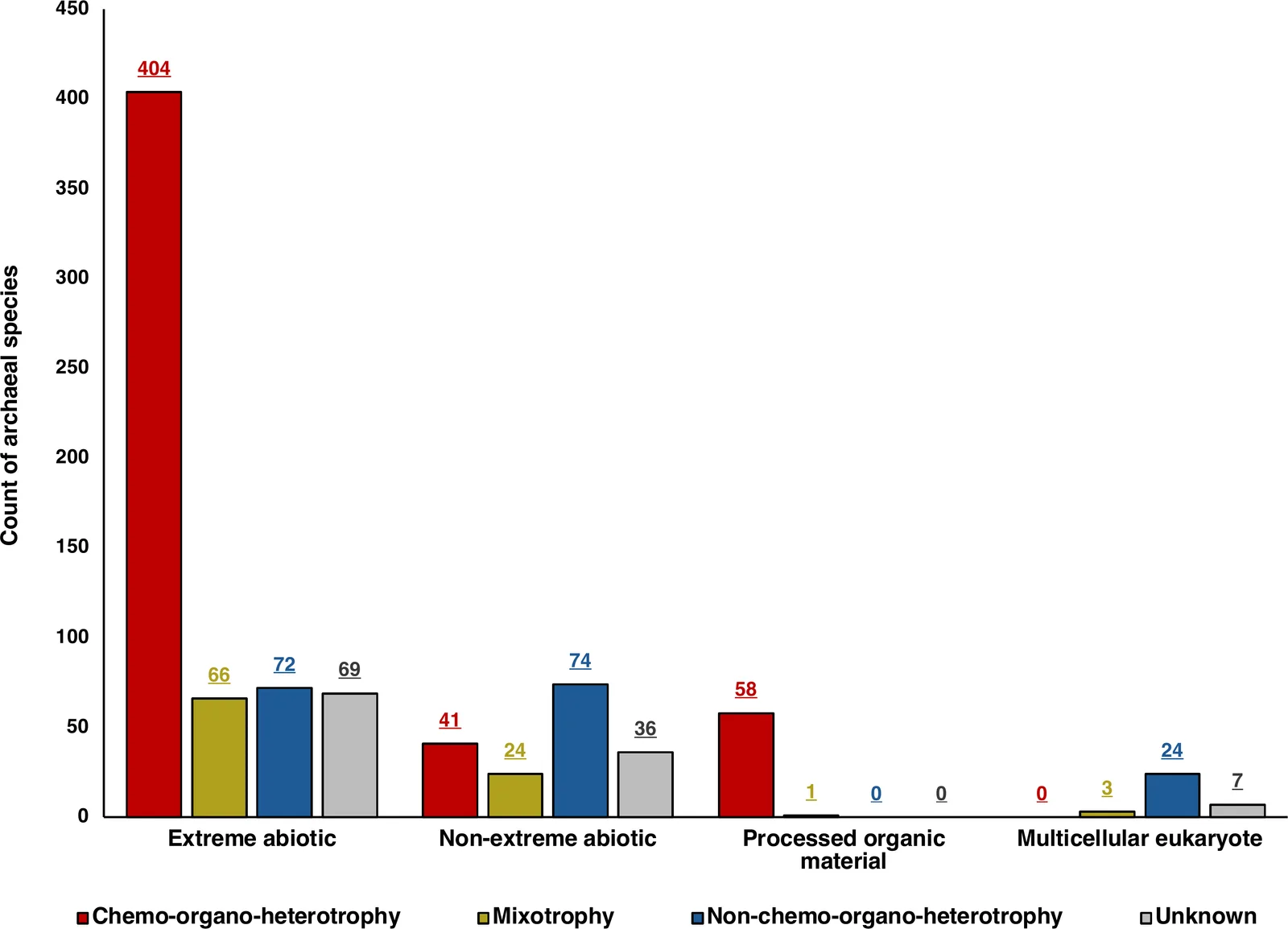
The distribution of archaeal metabolic annotations distinguished by habitats. This figure encompasses 806 archaeal species from the NCBI taxonomy database. Each species underwent manual annotation, incorporating metabolic and environmental information obtained from literature reviews, as detailed in Materials and Methods (“Data set III: habitat and metabolic annotations of archaea”). The bar colors correspond to distinct metabolic annotations: chemo-organo-heterotroph (red), mixotrophic (yellow), non-chemo-organo-heterotroph (blue), and unknown metabolic lifestyles (gray). Archaeal species with unknown metabolism are predominantly uncultured or unclassified.

Given that the presence or absence of COH capability alone cannot explain the absence of pathogenicity in the isolated archaea, we asked what factors, in addition to metabolism, might also constrain the evolution of pathogenicity among these archaea. We hypothesized that habitat is one such factor; specifically, the habitats archaea occupy lack multicellular eukaryotic hosts they can inhabit and subsequently infect. To test this hypothesis, we investigated the relationship between the metabolic capabilities and habitats of 806 isolated archaeal species through manual literature reviews (see Materials and Methods, “Data set III: habitat and metabolic annotations of archaea”). Specifically, we categorized species habitats into four distinct types: extreme abiotic environments (e.g., high temperature, high salinity, and high acidity), non-extreme abiotic environments (e.g., soil, marine sediment, seawater, and rivers), environments associated with dead eukaryotic material (e.g., salted foods or animal hides), and environments within multicellular eukaryotic hosts (e.g., animal guts). Figure 4 shows the number of archaeal species having different metabolic annotations for different habitat categories. This result shows that COH-capable archaeal species are predominantly free-living and do not inhabit multicellular eukaryotic hosts. Specifically, 470 (about 79%) of the COH-capable species inhabit environments characterized by extreme conditions, which do not allow most multicellular eukaryotic organisms to inhabit (Fig. 4). Of the remaining COH-capable species, 65 (about 11%) were isolated from non-extreme abiotic environments, and 59 (about 10%) from dead eukaryotic material, such as salted or fermented food and animal hides. These 59 species are halophilic, suggesting that the preservation methods, such as salting or fermentation, likely facilitate their colonization of the processed eukaryotic material (32). Notably, only three COH-capable species—namely, *Methanosarcina barkeri*, *Methanobrevibacter smithii*, and *Methanobrevibacter ruminantium—*have been isolated from living eukaryotic hosts (Fig. 4). However, they appear to be exceptions that warrant further investigation, as we describe in Discussion. The majority of archaeal species isolated from living eukaryotic hosts are non-COH (Fig. 4). They are mostly methanogens that can use organic compounds as a source of energy but require inorganic compounds as sources of carbon or electrons and are, thus, non-COH (18, 33). This result indicates that archaeal metabolism is distributed differently, depending on their habitats: non-COH archaeal species, while typically free-living, are the predominant archaeal residents inhabiting eukaryotes, whereas COH archaea are free-living and predominantly inhabit extreme environments.

**Fig 5:**
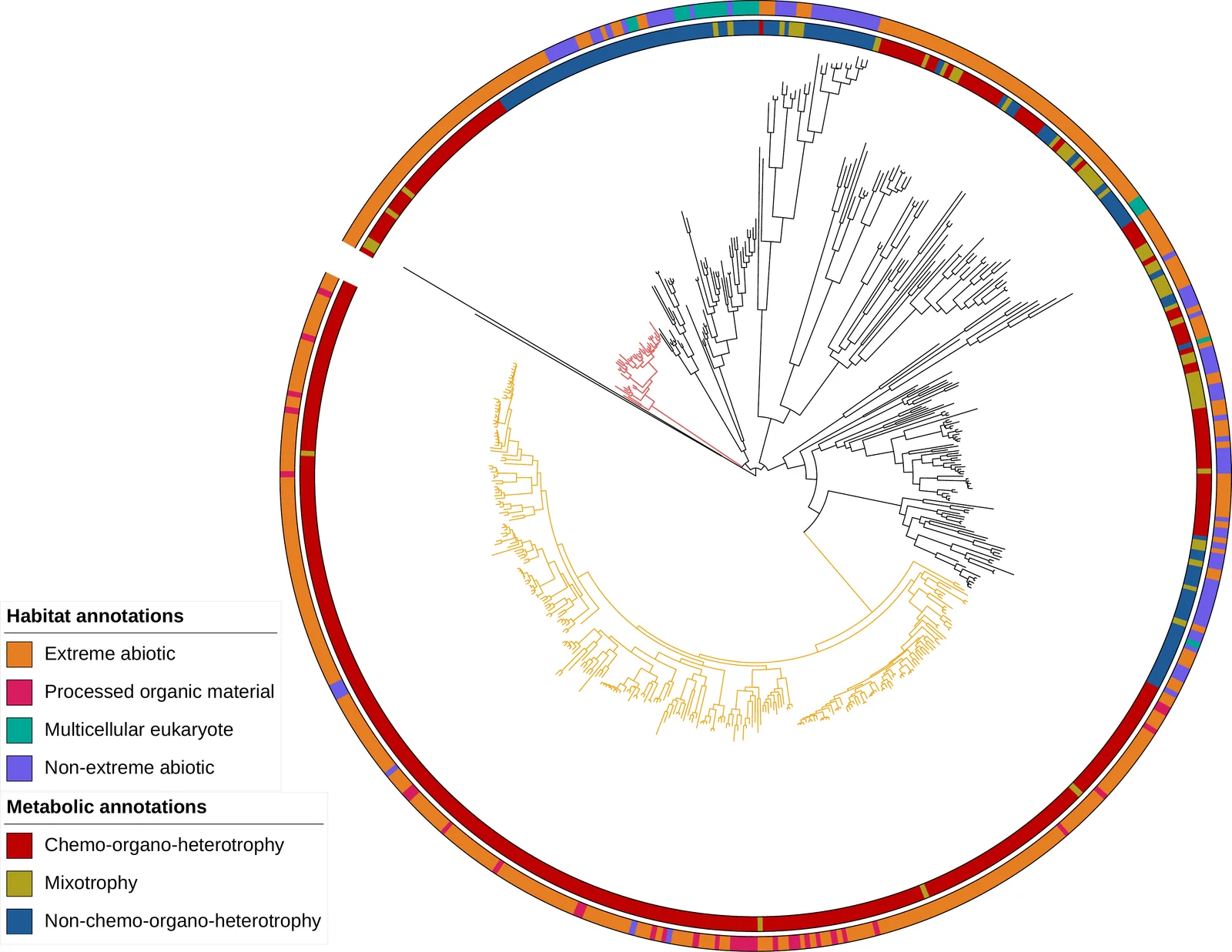
An archaeal tree containing 545 genomes annotated with metabolism (inner ring) and habitat (outer ring). Each tree tip corresponds to a GTDB representative genome, which may originate from an isolate, a single cell, or environmental samples. The tree was extracted from the Genome Taxonomy Database (34) and annotated using iTOL (31) as described in Materials and Methods (“Data set IV: annotations of the archaeal phylogeny”). The colors of the inner ring represent the following metabolic annotations: chemo-organo-heterotrophy (red), non-chemo-organo-heterotrophy (blue), and mixotrophy (olive). The colors of the outer ring represent the following habitat annotations: extreme abiotic (orange), non-extreme abiotic (purple), processed organic material (deep pink), and multicellular eukaryote (turquoise). Colored branches correspond to Halobacteria (yellow) and Thermococci (red) lineages. Genomes assigned an “unknown” tag for either metabolic or habitat annotation were pruned from the tree.

The preceding analysis assumes that each archaeal species represents an independent observation; however, closely related species are not independent, as they share a common ancestor. This raises the possibility that the correlation between metabolism and habitat is driven by shared ancestry rather than reflecting a direct relationship between the two traits. To investigate whether this correlation could be explained solely by shared ancestry, we mapped metabolic and habitat annotations onto an archaeal phylogeny from the Genome Taxonomy Database (34) using iTOL (31) as described in Materials and Methods (“Data set IV: annotations of the archaeal phylogeny”). The annotated phylogeny (Fig. 5) shows that metabolism and habitat types are scattered throughout the tree, suggesting that shared ancestry does not explain this correlation.

Given that the above results reflect a direct correlation between metabolism and habitat, we sought to test whether these traits also evolve in a correlated manner. To test this, we created a Markovian model in which the evolution of metabolism is potentially correlated with that of habitat (model D) and compared it with a nested model in which metabolic and habitat evolution are uncorrelated (model E), as described in Materials and Methods (“Statistical analysis of metabolism and habitat evolution”). Model D assumes four states, distinguishing either extremophile or non-extremophile and either COH capable or non-COH, and involves eight parameters representing transition rates between these states. Model E assumes that the transition rates between extremophile and non-extremophile habitat preference states are independent of metabolic states, and vice versa. Fitting these models to the archaeal phylogeny, we found that model D outperforms model E (Table 2), indicating that the metabolism and habitat preference of archaea evolve in a correlated manner.

**TABLE 2:** The model performance results comparing models D and E^*b*^

Model^*a*^	lnL	AIC	AIC wt^*c*^	*χ* ^2*d*^	*P*-value^*d*^
Model D	−235.4	486.8	100%		
	(−198.3)	(412.7)	(100%)		
Model E	−253.2	514.4	0%	35.6	3.5 *×* 10*^−^*^7^
	(−214.4)	(436.8)	(0%)	(32.1)	(1.8 *×* 10*^−^*^6^)

^*a*^ Model E is nested within model D (see Materials and Methods, “Statistical analysis of metabolism and habitat evolution”).

^*b*^ Values are shown for the “state-defined archaeal tree”; values in parentheses beneath correspond to the “clade-pruned archaeal tree.”.

^*c*^ AIC weights estimate the relative plausibility of each model.

^*d*^ Test statistics (*χ*^2^) and *P*-values from likelihood-ratio tests comparing models D and E.

However, the above results may not reflect a phylogeny-wide pattern, especially if certain clades are over-represented in a Markovian state—clades containing a disproportionately large number of species annotated with that state. To assess whether each state is distributed evenly across the archaeal phylogeny, we counted the number of annotated archaea in each taxonomic class (see Materials and Methods, “Data set IV: annotations of the archaeal phylogeny”). We found that Halobacteria and Thermococci were over-represented in the COH-capable extremophile state, as shown in Fig. 5 (for full counts, see Table S4 in the supplemental materials).

This result prompted us to investigate whether the correlated evolution result is robust to the over-representation of COH-capable extremophiles in Halobacteria and Thermococci. Specifically, we fitted models D and E to a pruned archaeal phylogeny that excludes these clades (for further details, see Materials and Methods, “Data set IV: annotations of the archaeal phylogeny”). Comparing these two models, we found that model D significantly outperforms model E, consistent with the result on the full archaeal phylogeny (Table 2). This indicates that the correlation between metabolism and habitat is not driven by the over-representation of COH-capable extremophiles in Halobacteria and Thermococci.

Taken together, these findings indicate a correlated evolution of metabolism and habitat preference across isolated archaeal lineages, which may help explain the conspicuous absence of pathogenic archaea. Specifically, COH-capable lineages are predominantly free-living and occupy extreme environments that are largely devoid of multicellular eukaryotes, which limits their opportunities to colonize and cause infectious disease in potential eukaryotic hosts.

## DISCUSSION

The results presented above indicate that the ability to perform COH is a likely metabolic prerequisite for bacterial pathogenicity. While COH metabolism is widespread among isolated archaea, it is predominantly found in the species living in extreme environments and is notably absent from those that consistently colonize living eukaryotic multicellular hosts, with a few exceptions discussed below. These findings lead us to hypothesize that the conspicuous absence of archaeal pathogens may be explained by a combination of lack of metabolic capability and ecological opportunity, rather than the lack of metabolic capability alone as previously suggested (7, 28). Specifically, COH-capable archaea, despite having the metabolic prerequisite for pathogenicity, lack the ecological opportunity to interact with and inhabit multicellular eukaryotic hosts, whereas non-COH archaea have not been shown to possess this metabolic prerequisite.

We speculate that COH may be a metabolic prerequisite for pathogenicity because it minimizes resource constraints on pathogen proliferation within host tissues. COH-capable microbes are, by definition, able to acquire energy, electrons, and carbon from organic compounds, which are abundant in host tissues (35–37). In contrast, non-COH microbes must acquire one or more of these resources from inorganic compounds, which may be scarce or absent in host tissues. This scarcity of any of the essential resources within host tissues is expected to constrain the proliferation of host-associated microbes (35, 38–40). For example, host-associated chemo-litho-heterotrophic microbes, which include hydrogenotrophic methanogens, can obtain carbon from host organic compounds but depend on inorganic electron donors, such as hydrogen, which may be scarce in host tissue. However, this scarcity would not constrain the proliferation of chemo-organo-autotrophic microbes because the carbon dioxide and organic compounds they require are abundant in host tissue. As such, the constraints acting on their proliferation within the host remain unclear, although one possibility is the energetic cost of carbon fixation. Overall, non-COH microbes would generally encounter greater constraints on their proliferation within host tissues than COH-capable microbes, which can obtain all their required resources directly from host tissues.

A potential limitation of our hypothesis is that it is derived from the analysis of isolated archaea, and its applicability to uncultured archaea remains to be tested. Given that the vast majority of archaea remain uncultured, future isolates could challenge our hypothesis, especially if COH-capable archaea are later identified as typical residents of multicellular eukaryotes. Nevertheless, our hypothesis, based on the comprehensive analyses of thousands of isolated archaea, offers an explanation for the absence of pathogenicity in isolated archaea and, by extension, a basis for inferring the metabolism of uncultured archaea from their habitat. Specifically, uncultured archaea inhabiting extreme environments are likely COH-capable, while those inhabiting eukaryotic hosts are likely non-COH.

The potential exceptions to our hypothesis are the three mixotrophic (i.e., capable of both COH and non-COH) archaeal species that have been isolated from eukaryotic hosts (Fig. 4). Although they may exemplify host-associated archaea capable of COH, it is currently unclear whether they constitute genuine counterexamples to the observed segregation of archaeal metabolism by habitats. Specifically, two of these species, *Mbr. smithii* and *Mbr. ruminantium*, are chemo-litho-heterotrophic (non-COH), sourcing their electrons from molecular hydrogen (41, 42), and COH, sourcing their energy and electrons from organic formate and their carbon from acetate (41, 42). However, they cannot sustain COH-based growth when formate is the sole source of energy and electrons, in the absence of hydrogen (43, 44), suggesting that hydrogen is the preferred electron donor. Notably, both *Mbr. smithii* and *Mbr. ruminantium* typically reside within the digestive tract of animals (41, 42), which is rich in molecular hydrogen produced primarily by fermentative microbes (45). Therefore, when these *Methanobrevibacter* species inhabit the digestive tract, they may primarily perform non-COH, sourcing energy and electrons from molecular hydrogen, with COH metabolism serving as an auxiliary metabolism. The third mixotrophic species, *Msa. barkeri*, is capable of COH metabolism, sourcing energy, electrons, and carbon from methanol, methylamines, or acetate (42, 46–48). This species can also perform non-COH metabolism, either chemo-litho-autotrophy or chemo-litho-heterotrophy, sourcing electrons and energy from hydrogen and carbon from either carbon dioxide or methanol (42, 46, 47, 49). Notably, one study reports an isolation of *Msa. barkeri* from the rumen of a cow (48), thus providing a potential exception to our hypothesis. However, no subsequent isolations of *Msa. barkeri* from a rumen have been reported since. Moreover, Msa. barkeri has been isolated multiple times from non-host-associated environments, such as anaerobic aquatic habitats (47, 49, 50). This repeated isolation from non-host-associated environments is consistent with that of other *Methanosarcina* species, which have been isolated primarily from aquatic habitats (42). Given the single isolation of *Msa. barkeri* from the rumen weighed against the repeated isolation of *Methanosarcina* species from non-host-associated environments, whether *Msa. barkeri* is a stable inhabitant or a transient member of the rumen is unclear. Taken together, these findings suggest that these three species may not constitute a direct counterexample to our observation that COH-capable archaea seldom reside stably within multicellular eukaryotic hosts.

Our hypothesis is not undermined by the abundance of archaea that inhabit eukaryotic hosts and consume organic substrates, since mere consumption of organic substrates does not constitute COH, which is defined as the ability to use organic substrates as the source of electrons, carbon, and energy. For example, species of *Methanosphaera* and *Methanomassiliicoccus* are frequently isolated from eukaryotic hosts and are capable of consuming organic substrates (42, 51, 52). However, they depend on hydrogen gas as the source of electrons and are thus not COH organisms.

Our hypothesis predicts that if there were archaea capable of both COH and host colonization, they would have the potential to evolve pathogenicity toward their hosts. This prediction finds an illustrative parallel in the archaeon *Candidatus* Nanohaloarchaeum antarcticus, which can exploit its archaeal host, *Halorubrum lacusprofundi*, through a process analogous to pathogenicity (53). Specifically, *Ca*. Nha. Antarcticus attaches to its archaeal host, where it may invade its host cell and induce host cell lysis (53). Notably, previous studies indicate that the genome of *Ca*. Nha. antarcticus supports a COH-capable metabolism (54), which our findings suggest is a metabolic prerequisite of pathogenicity toward eukaryotes. In addition, both *Ca*. Nha. Antarcticus and *H. lacusprofundi* inhabit Rauer 1 Lake, an Antarctic hypersaline lake (53, 54), which would provide *Ca*. Nha. antarcticus with ample opportunity to adapt to colonize its host. Interestingly, the extreme salinity does not appear to preclude *Ca*. Nha. antarcticus from exploiting its host, which suggests that extreme conditions *per se* may not preclude archaea from pathogenicity. Therefore, if COH-capable archaea, which typically live in extreme environments, were provided ample opportunity to interact with multicellular eukaryotic organisms, they might evolve pathogenicity.

While our hypothesis is grounded in information about thousands of bacterial and archaeal species, the existing metabolic constraint hypotheses are based on information about a handful of well-known bacterial pathogens and archaeal species (7, 10, 28). Therefore, the applicability of these existing hypotheses beyond those specific species needs to be considered. Indeed, Cavicchioli and Curmi question Martin’s hypothesis on this ground (28, 55). Martin (28) has hypothesized that archaea are non-pathogenic because their unique cofactor requirement prohibits them from exploiting cofactors from their host (28). To counter this hypothesis, Cavicchioli and Curmi highlight that many methanogenic archaea synthesize their own cofactors while inhabiting their host, arguing that a unique cofactor requirement does not restrict archaea from host colonization (55). Moreover, they argue that many bacterial pathogens exploit various resources beyond cofactors from their host, so that the inability to exploit cofactors from their host does not necessarily preclude archaea from pathogenicity (55).

Extending Cavicchioli and Curmi’s discussion (55), we consider the other metabolic constraint hypotheses in light of the diverse metabolic capacities of bacterial pathogens and archaeal species covered by the present study. Valentine has hypothesized that archaea are non-pathogenic because they cannot cope with fluctuating environmental conditions, particularly resource availability, during infection (7). This hypothesis does not appear to align with the adaptability observed in certain species of methanogenic archaea. Namely, species within the *Methanosarcina* genus demonstrate the ability to obtain carbon and energy from various compounds, exhibit a broad niche range, and thrive in environments with fluctuating resource availability (42, 56, 57). This adaptability within the *Methanosarcina* genus suggests that the capability to thrive under fluctuating environmental conditions, including variations in resource availability, is not universally absent in archaea.

The metabolic constraint hypothesis proposed by Abedon posits that the ability to exploit complex organic compounds such as glucose—where the complexity can be measured by the number of carbon atoms in a compound—is a prerequisite for pathogenicity and that this prerequisite is lacking in the archaea inhabiting non-extreme environments (10). Abedon’s hypothesis is largely supported by our data set (Data set III), which indicates that archaeal species inhabiting non-extreme environments are rarely capable of metabolizing complex organic compounds. Specifically, of the 493 archaeal species capable of metabolizing organic compounds containing at least six carbon atoms, 404 (about 82%) are isolated from extreme environments, and none from multicellular eukaryotic hosts. As a corollary, all 27 archaeal species isolated from eukaryotic hosts consume organic compounds containing fewer than six carbon atoms. These results mean that the ability to metabolize complex organic compounds in archaea is differentially distributed depending on their habitats, highlighting a similarity between Abedon’s hypothesis and ours. However, Abedon’s hypothesis does not appear to fully agree with the known metabolism of pathogenic bacteria. Namely, *Legionella pneumophila*, *Francisella tularensis*, and *Pseudomonas syringae*, although capable of consuming complex organic compounds, are dependent on simple organic compounds, such as cysteine and pyruvate, for their growth during infection (58–62). By contrast, this dependence constitutes COH and is, therefore, compatible with our hypothesis.

Finally, future work that extends our findings to encompass uncultured archaea would require approaches involving theoretical predictions, such as inference based on genome sequences, for which this study can provide reference data.

In summary, our study reveals a potential link between the conspicuous absence of archaeal pathogenicity and the environmental separation of archaeal chemo-organo-heterotrophy, raising a question for future investigation: why are no chemo-organo-heterotrophic archaea consistent residents of eukaryotic hosts?

## MATERIALS AND METHODS

### Data set I: metabolic and pathogenic annotations of bacterial isolates

To identify the core metabolic abilities of bacterial pathogens, a data set (referred to as “Data set I” with rows where Dataset_source = Dataset_I; available as supplemental data, “Data set S1”) consisting of the metabolic, habitat, and pathogenic annotations of 3,125 bacterial isolates was created as follows. The Integrated Microbial Genomes with Microbiome Samples (IMG/M) database (29) was accessed in August 2023 to extract the metadata of 110,457 bacterial genome assemblies for the following 12 metadata categories: Biotic_Relationships, Diseases, Ecosystem, Ecosystem_Category, Ecosystem_Subtype, Ecosystem_Type, Energy_Source, Habitat, Isolation, Metabolism, Phenotype, and Specific_Ecosystem. The downloaded metadata were processed to extract information about the habitat, pathogenicity, and metabolic abilities of bacteria, as follows.

The metadata of all bacterial genome assemblies were combined to generate 10,996 unique pairs of a metadata category and metadata text (e.g., category = Phenotype, text = *“*Biodegradation, Non-Pathogen*”*). Each category-text pair was manually inspected and assigned zero or more of the habitat, pathogenic, and metabolic annotation tags, as described in the following paragraphs (for the metadata category Isolation, the unique metadata texts were numerous [over 8,000], so they were pre-filtered by retaining only those containing at least one of the following keywords: blood, lungs, culture, heart, bacteremia, infection, heterotroph, healthy, normal, CSF, and disease). Category-text pairs assigned no annotation tags were excluded from further analysis. The result of the annotation tag assignment is referred to as “annotated metadata list” and is available as supplemental data, “Data set S2.” Habitat annotation tags, Host or Free-living, were assigned when a category-text pair states that a bacterium was sampled from living multicellular eukaryotic hosts or existed as a free-living entity, respectively.

Pathogenicity was defined here as the ability to infect a multicellular eukaryotic host (e.g., animals, plants, and insects) and cause disease. The Pathogen tag was assigned when a category-text pair states that a bacterium is a pathogen or when the disease the bacterium causes is specified under the Diseases category. The Non-pathogen tag was assigned when a category-text pair states that a bacterium was isolated from a healthy patient, is part of the host’s normal microbiome, does not inhabit any eukaryotic multicellular host, is explicitly described as non-pathogenic, or is associated with no disease (specifically, when the metadata text under the Diseases category is not blank and explicitly states “none”).

Metabolic annotation tags were assigned when a category-text pair states that a bacterium possesses the following abilities to acquire one of the three resources: for energy acquisition, Chemotrophy or Phototrophy (chemicals or light, respectively); for electron acquisition, Organotrophy or Lithotrophy (organic or inorganic compounds, respectively); and for carbon acquisition, Heterotrophy or Autotrophy (organic compounds or carbon dioxide, respectively). When multiple metabolic annotation tags were assigned, they were combined into one tag, such as Chemo-organo-heterotroph. Some tag assignments were flagged as inconclusive when metadata texts did not allow straightforward interpretations. These flagged cases underwent further manual examination as described later.

Using the result of the above annotation-tag assignment, each genome assembly was annotated by taking the union of all annotation tags assigned to the category-text pairs of its metadata.

To integrate information across different genome assemblies of an identical bacterial isolate, assemblies sharing an identical NCBI taxonomy ID were collapsed into an “isolate,” with isolate-level annotation defined as the union of all annotation tags of respective assemblies. The metabolic annotation tag of an isolate was categorized into one of the following groups when it specified the ability to acquire all three resources: *c*hemo-organo-heterotroph, non-chemo-organo-heterotroph, or mixotrophy. The non-COH category was defined as any combination of metabolic annotation tags that do not include all of chemotrophy, organotrophy, and *heterotrophy* (e.g., photo-litho-heterotrophy and chemo-organo-autotrophy). The mixotrophy category was defined as the possession of chemotrophy, organotrophy, heterotrophy, and at least one of phototrophy, lithotrophy, or autotrophy.

The isolates with metabolic annotation tags that do not specify the ability to acquire all three resources were flagged as inconclusive. These isolates were manually examined by reviewing the literature on them (the result of this literature review is included in supplemental data “Data set S2”). In addition, the pathogenic annotation tag of some cyanobacteria isolates was manually corrected to non-pathogenic: although these cyanobacteria can cause illness in eukaryotes by contaminating water with toxins, they are not known to infect and cause any diseases in eukaryotic hosts (63) and therefore are classified as non-pathogenic under our definition of pathogenicity.

To ensure the valid taxonomic identification of isolates, each isolate’s NCBI taxonomy ID was mapped to species ID, NCBI taxonomy, and organism name using the Genome Taxonomy Database (GTDB) (64). Isolates were discarded from our data set if their NCBI taxonomy ID was mapped to multiple distinct species IDs or if their genus or species names were enclosed in brackets (or both), as these situations suggest errors, obsolescence, or ongoing revision. The isolates that had either no metabolic or pathogenic annotation tag were removed from the data set. The resulting data set containing 3,125 isolates, referred to as Data set I (Dataset_source = Dataset_I), is available as supplemental data.

The resulting isolate-level annotations were integrated into species-level annotations, as follows. The habitat annotation tags of each species were generated by taking the union of the habitat annotation tags of respective isolates. The pathogenic annotation tag of a species was set to pathogenic if at least one of its isolates was annotated as pathogenic or non-pathogenic if none of its isolates was annotated as *pathogenic* and at least one isolate was annotated as non-pathogenic (in all other cases, it was set to unknown). The metabolic annotation tag of a species was set as chemo-organo-heterotrophy, non-chemo-organo-heterotrophy*,* or mixotroph based on the union of all metabolic annotation tags of its isolates as described above. The remaining cases were set to unknown, which consists of incomplete metabolic tag combinations, such as chemo-organotroph.

### A bacterial phylogeny

To estimate the rates of evolution of metabolism and pathogenicity (see below, “Statistical analysis of metabolism and pathogenicity evolution”), a phylogenetic tree comprising 1,718 eukaryotic, bacterial, and archaeal isolates (tips) and their NCBI taxonomy IDs was downloaded from the MicrobesOnline database (30) in July 2021. Each tree tip represents a sequenced genome that was derived from an isolate. To create a bacterium-only phylogeny for downstream analyses, we pruned all tips whose NCBI taxonomy IDs correspond to archaeal (*n* = 93) and eukaryotic (*n* = 80) taxa using the “ape” package (65) in R (https://www.R-project.org/). To refine the taxonomic identification of the isolates, those isolates whose NCBI taxonomy IDs were no longer included in the NCBI taxonomy database were filtered out (*n* = 25), resulting in 1,520 bacterial isolates. Some branches in our phylogeny have a length of zero, indicating that any evolutionary change was too small to detect. Therefore, to account for this minimal evolutionary change, we assigned those branches a length of 1 *×* 10*^−^*^8^ substitutions per site. The resulting phylogenetic tree, referred to as “bacterial tree,” is available as supplemental data, “Data set S3.”

### Data set II: annotations of the bacterial phylogeny

To annotate the above bacterial phylogeny, a data set (referred to as “Data set II”; available as supplemental data, “Data set S4”) consisting of the metabolic and pathogenic annotations for 1,520 bacterial isolates included in the phylogeny was created as follows. For each isolate in the MicrobesOnline tree, its corresponding NCBI Taxonomy ID and isolate name (tree tip label) were extracted. Each isolate was assigned a metabolic and pathogenic annotation by extracting its annotation tags from Data set I. For isolates absent in Data set I, we assigned a metabolic and pathogenic annotation by conducting manual literature reviews. The resulting additional annotated isolates were added to Data set I, increasing the number of annotated isolates and species by about 30% (to 4,235 and 1,249, respectively). Isolates that had either no metabolic or pathogenic annotation tag were excluded from Data set I but kept in Data set II for tree visualization (described in the following paragraph). The source of each metabolic and pathogenic annotation is described in Data set I under columns “Metabolic_evidence” and “Pathogenic_evidence,” respectively. The source of the annotation is also stated under the “Dataset_source” column by designating Dataset_I if an isolate’s metabolic and pathogenic annotations were sourced from IMG/M, Dataset_II if annotations were sourced from literature reviews, or Dataset_I & Dataset_II if annotations were sourced from both IMG/M and literature reviews.

To visualize the bacterial phylogeny annotated with metabolism and pathogenicity tags, the annotations of each isolate were assigned to its corresponding tree tip using the Interactive Tree Of Life tool (iTOL; https://itol.embl.de/).

To perform the statistical analyses described below (see “Statistical analysis of metabolism and pathogenicity evolution”), we pruned the annotated bacterial phylogeny as follows. We removed tree tips that lacked a metabolic or pathogenic annotation, using “ape” in R. Each isolate present in the pruned phylogeny is labeled Visualization & Statistical analysis in the “usage” column of Data set II; isolates absent from the pruned phylogeny are labeled Visualization alone. The pruned phylogeny consists of 1,462 tips, which we refer to as a “state-defined bacterial tree.”

For each isolate, we assigned one of the following three states describing metabolism and pathogenicity: capable of chemo-organo-heterotrophy (which includes mixotrophy) and pathogen (state 1), capable of chemo-organo-heterotrophy and non-pathogen (state 2), and incapable of chemo-organo-heterotrophy (or exclusively non-COH) and non-pathogen (state 3). While our two binary traits yield four possible state combinations, our data set lacks the fourth state, that is, pathogenic bacteria that were exclusively non-COH. The state counts and proportions, reported in the supplemental materials (Table S3), were as follows: state 1, 805 (54.0%); state 2, 568 (38.1%); and state 3, 119 (8.0%). These metabolic-pathogenic states are recorded under the “Isolate_Markovian_state” column of Data set II.

The observed predominance of states 1 and 2 may reflect an over-representation of closely related isolates within particular clades, which could bias our analyses, as any detected correlated evolution may be solely driven by those clades rather than the entire phylogeny. Therefore, to assess whether our analyses were biased by an over-representation of particular clades, we examined the distribution of isolates across phyla for each state and reported these distributions in the supplemental materials (Table S2), where Pseudomonadota and Bacillota were identified as over-represented in states 1 and 2.

To test whether our analyses detected correlated evolution within Pseudomonadota and Bacillota rather than across the entire phylogeny, we pruned the “bacterial tree” to exclude these two clades and then repeated the same analyses on this tree. This repeated analysis on a pruned tree serves as a robustness check for the potential bias introduced by the over-representation of Pseudomonadota and Bacillota. Removing these phyla resulted in a tree with 592 tips, referred to as the “clade-pruned bacterial tree.” The state counts and proportions for this tree, reported in the supplemental materials (Table S3), were as follows: state 1, 245 (41.4%); state 2, 262 (44.3%); and state 3, 85 (14.4%).

### Statistical analysis of metabolism and pathogenicity evolution

To estimate the rates of evolution of metabolism and pathogenicity, we fitted a continuous-time Markov model (66) to our state-defined bacterial tree annotated with isolate-level metabolic-pathogenic states using the “castor” package (67) in R. To allow for the correlated evolution of metabolism and pathogenicity, we created a four-state model with eight evolutionary rate parameters, which we refer to as model A (Fig. 2). To determine whether the evolution of metabolism and that of pathogenicity are correlated, we created two nested models, models B and C, to compare with model A. In model B, pathogenic and metabolic evolution are made independent by setting the following pairs of evolutionary rate parameters equal: *Q*_1_ = *Q*_7_, *Q*_2_ = *Q*_8_, *Q*_3_ = *Q*_5_, and *Q*_4_ = *Q*_6_. In model C, the number of states is effectively reduced to three by fixing the rates of evolution to and from the non-COH pathogen state to zero: *Q*_3_ = *Q*_4_ = *Q*_7_ = *Q*_8_ = 0. For each model, a log-likelihood and akaike information criterion (AIC) value were calculated and used for model comparison. To obtain the confidence interval of the rates of metabolic and pathogenic evolution, we conducted 1,000 parametric bootstraps on each estimated rate using the “castor” package. To assess whether the over-representation of chemo-organo-heterotrophic states (i.e., states 1 and 2) belonging to the Pseudomonadota and Bacillota phyla biases our results, we repeated the analyses using a pruned phylogeny which excludes these clades.

### Data set III: habitat and metabolic annotations of archaea

To examine the co-distribution of metabolic abilities and habitat of archaea, a data set (referred to as “Data set III”; available as supplemental data, “Data set S5”) containing the habitat and metabolic annotations of archaeal isolates and species was created based on manual literature reviews. These manual reviews were deemed necessary since the existing databases, such as IMG/M, contained a small number of archaeal entries (e.g., 21 times fewer than bacteria in IMG/M) and frequently lacked metadata for archaea. As these annotations were derived from manual reviews, primarily of experimental studies, the data set largely comprises isolated archaea for which metabolic capabilities and habitats have been experimentally characterized.

To create this data set, a list of 837 formally named archaeal species was extracted from the NCBI Taxonomy Database (1) between 2022 and 2023. Each species name was queried on the NCBI Taxonomy Browser to extract corresponding isolate names, resulting in a list of 5,248 isolates.

To annotate these isolates regarding their metabolism and sampled environment, each isolate’s name was queried on the following databases to retrieve linked literature: the NCBI Taxonomy Browser (1), the Japanese Collection of Microorganisms (JCM; http://www.jcm.riken.jp/), American Type Culture Collection (ATCC; http://www.atcc.org/), Deutsche Sammlung von Mikroorganismen und Zellkulturen GmbH (DSMZ; https://www.dsmz.de), and Bacterial Diversity Metadatabase (Bacdive; (68)). Where linked literature was absent or lacked sufficient information, we conducted manual literature searches about all of these isolates. The references used for the annotation are provided under the “Source” column in Data set III. Additionally, we specified whether each reference was sourced from peer-reviewed articles (“sourced from article”), any NCBI database (“sourced from NCBI database*”*), or sourced from a culture database (“sourced from culture database*”*), under the “Source_category” column. The literature was manually processed to extract relevant information about the isolates, as follows.

For habitat annotation, an isolate was assigned one of the following tags: Multicellular eukaryote, Processed organic material, Extreme abiotic, or Non-extreme abiotic, which respectively mean that an isolate was sampled from living eukaryotic hosts, processed organic material such as salted food or animal hides, abiotic environments with extreme conditions (namely, extreme temperature, pH, pressure, hydrocarbon levels, or salinity), or abiotic environments with non-extreme conditions. To add context to the habitat annotation, we included comments under “Habitat_comment,” which describe the site in which each archaeon was sampled (e.g., sulfur-rich acidic edge of a solfataric hot spring). These comments were then manually categorized into specific labels (e.g., goat, extreme salinity, etc.) under “habitat_type.”

For metabolic annotation, an isolate was assigned one or more of the following tags: “chemo-organo-heterotroph,” “non-chemo-organo-heterotroph,” “mixotroph,” and “unknown.” To provide context to the metabolic annotation, we listed every compound each archaeon was described to metabolize for energy, electrons, or carbon under the “Metabolic_substrates” column. The number of compounds each archaeon metabolized is provided under the “Substrate_diversity” column. We also indicate whether each archaeon could metabolize a complex carbon compound (defined as a compound containing six or more carbon atoms) or not (designated as complex and simple, respectively) under the “substrate_complexity” column.

In cases where adequate information about the metabolism and sampled site of an isolate was unavailable, the isolate’s metabolism and habitat were annotated as unknown.

The resulting isolate-level annotations were integrated into species-level annotations in the same manner as Data set I. For research beyond this work, our database also provides, when available, genomic metadata extracted from NCBI: genome assembly level (e.g., Complete Genome, Scaffold, or Contig), genome size (base pairs), and NCBI genome assembly accession (retrieved from either GenBank or RefSeq). The accession is also used to create Data set IV (described below under “Data set IV: annotations of the archaeal phylogeny”), a subsequent data set required for the statistical analyses described in the following sections. Briefly, the accession serves as a key linking Data set IV to Data set III. This resulted in a novel database containing literature-supported information about 806 archaeal species (Data set S5).

### An archaeal phylogeny

To estimate the rates of evolution of metabolism and habitat among archaea (see below, “Statistical analysis of metabolism and habitat evolution”), we downloaded a phylogenetic tree consisting of 6,968 archaeal genomes (tips) from the Genome Taxonomy Database (GTDB) in February 2025 (34). Each tip corresponds to a GTDB representative genome, which may originate from an isolate, a single cell, or environmental samples (metagenomes), unlike the bacterial phylogeny, which consists only of isolate-derived genomes. We pruned the tree to retain only genomes present in Data set III, resulting in a phylogeny with 642 tips. This pruned tree, hereafter referred to as the “archaeal tree,” is provided as supplemental data (“Data set S6.

### Data set IV: annotations of the archaeal phylogeny

For downstream analyses of metabolism and habitat evolution among archaea, we annotated the 642 tips of the archaeal tree with their GTDB taxonomy, metabolic, habitat, and Markovian states (Data set S7), as follows.

To annotate each tip with its GTDB taxonomy, spanning from species to phylum, we extracted the corresponding metadata from GTDB in February 2025. Each tip is labeled with its GTDB genome accession, which has a GTDB-specific prefix (RS_ for RefSeq or GB_ for GenBank), followed by the corresponding NCBI database prefix (GCF_ for RefSeq or GCA_ for GenBank) and its NCBI numeric identifier.

To annotate each tip with its metabolic and habitat annotations, tip labels were matched to the NCBI accessions in Data set III on the shared numeric assembly identifier, regardless of the NCBI database prefix. These database prefixes denote different curated versions of the same genome assembly rather than distinct assemblies.

To visualize the distribution of metabolic and habitat annotations in the archaeal phylogeny, we mapped the tip annotations to the tree using iTOL (https://itol.embl.de/).

To perform the statistical analyses described below (see “Statistical analysis of metabolic and habitat evolution”), we pruned the archaeal phylogeny to retain only tips with complete metabolic and habitat annotations, as their states are derived from experimental observations rather than model estimates. This pruned phylogeny, hereafter referred to as the “state-defined archaeal tree,” consists of 545 tips.

For each tip, we assigned one of the following four states describing metabolism and habitat: capable of chemo-organo-heterotrophy (which includes mixotrophy) and extremophile (state 1, *n* = 395), capable of chemo-organo-heterotrophy and non-extremophile (state 2, *n* = 35), incapable of chemo-organo-heterotrophy (i.e., exclusively non-COH) and extremophile (state 3, *n* = 45), and incapable of chemo-organo-heterotrophy and non-extremophile (state 4, *n* = 70).

We distinguish “extremophile” and “non-extremophile” broadly to reflect whether a habitat was considered accessible to most multicellular eukaryotes. The extremophile state was assigned to tips that were annotated with either the “extreme abiotic environment” or “processed organic material” habitat tags. While the “processed organic material” habitat tag includes salted materials such as salted hides and foods that humans may handle, the salinity of these materials is generally too extreme to support most multicellular eukaryotes as a sustained habitat. The “non-extremophile” state was assigned to tips annotated with either a “non-extreme abiotic environment” or “multi-cellular eukaryote” habitat tag. These metabolic-habitat states and their corresponding ID (i.e, 1, 2, 3, 4) are recorded under the “Joint_state” and “Joint_state_integer” columns of Data set IV, respectively.

The observed predominance of state 1 (*n* = 395 of 545) may reflect an over-representation of closely related genomes within particular clades, which could bias our analyses (as described above under “Data set II: annotations of the bacterial phylogeny”). To assess this, we examined the distribution of genomes across classes for each state (Table S4), identifying Halobacteria and Thermococci as over-represented in state 1.

To test whether the detected correlated evolution was driven by the over-representation of Halobacteria and Thermococci or reflects a pattern across the entire phylogeny, we pruned the state-defined archaeal tree to exclude these two clades and repeated the analyses on this pruned tree. This repeated analysis on a pruned tree serves as a robustness check for the potential bias introduced by the over-representation of Halobacteria and Thermococci. The resulting tree consists of 235 tips and is referred to as the “clade-pruned archaeal tree.” The counts and proportions of each Markovian state for this tree are shown in Table S5.

### Statistical analysis of metabolism and habitat evolution

To estimate the rates of metabolic and habitat evolution, we used the statistical framework described above (see “Statistical analysis of metabolism and pathogenicity evolution”) and applied it to an archaeal phylogeny annotated with genome-level metabolic-habitat states. Specifically, we fitted a continuous-time Markov model (66) using the castor package (67), allowing for correlated evolution between metabolism and habitat with model D, which has a structure similar to that in Fig. 2), with eight rate parameters. To test whether metabolism and habitat evolve independently, we constructed a nested model (model E) in which independence between metabolic and habitat evolution is enforced by applying the same rate constraints as Model B in the bacterial analysis (see above, “Statistical analysis of metabolism and pathogen evolution”). To assess the robustness of the analysis to the over-representation of genomes assigned a chemo-organo-heterotrophic capable, extremophile state (i.e., state 1) that belong to Halobacteria and Thermococci classes (see Table S4 in supplemental material), we repeated the analyses on a pruned phylogeny that excludes these two clades.

## Data Availability

All data underlying the conclusions are provided as supplemental material.

## SUPPLEMENTAL MATERIAL

Data set S1 mbio.01185-26-s0001.txt Metabolic, habitat, and pathogenic annotations of bacterial isolates.

Data set S2 mbio.01185-26-s0002.txt Tags and their annotations.

Data set S3 mbio.01185-26-s0003.txt Bacterial tree in Newick format.

Data set S4 mbio.01185-26-s0004.txt Annotated tips of a bacterial phylogeny.

Data set S5 mbio.01185-26-s0005.txt Habitat and metabolic annotations of archaea.

Data set S6 mbio.01185-26-s0006.txt Archaeal tree in Newick format.

Data set S7 mbio.01185-26-s0007.txt Annotated tips of an archaeal phylogeny.

Supplemental legends mbio.01185-26-s0008.docx Legends for Data sets S1-S7 and Tables S1-S5.

Supplemental tables mbio.01185-26-s0009.docx Tables S1-S5.

ASM does not own the copyrights to Supplemental Material that may be linked to, or accessed through, an article. The authors have granted ASM a non-exclusive, world-wide license to publish the Supplemental Material files. Please contact the corresponding author directly for reuse.
