# Supplementary material for: Why are archaea not pathogenic? A hypothesis based on metabolism-habitat covariation
Source: mBio. 2026 Aug 21;17(9):e01185-26. doi: 10.1128/mbio.01185-26 (PMC13556234; doi:10.1128/mbio.01185-26)
Supplement: Supplementary file 1 [file mbio.01185-26-s0008.docx]

**Supplemental legends**

Daniel J. Harrison^a#^, Matthew S. Fullmer^b^, and Nobuto Takeuchi^a,b,c^

^a^School of Biological Sciences, University of Auckland, Auckland, New Zealand

^b^Research Centre for Complex Systems Biology, Universal Biology Institute, University of Tokyo, Meguro-ku, Tokyo, Japan

^c^Department of Biology, Faculty of Sciences, Kyushu University, Fukuoka, Japan

#Address Correspondence to Daniel J. Harrison, [dhar522@aucklanduni.ac.nz](mailto:dhar522@aucklanduni.ac.nz)

**Dataset S1–S7**

Dataset S1. A dataset containing the metabolic, habitat and pathogenic annotations of bacterial isolates.

Dataset S2. A list of tags and their annotations.

Dataset S3. A Bacterial tree in Newick format.

Dataset S4. A dataset containing the annotated tips of a bacterial phylogeny.

Dataset S5. A dataset of habitat and metabolic annotations of archaea.

Dataset S6. An archaeal tree in Newick format.

Dataset S7. A dataset containing the annotated tips of an archaeal phylogeny.

**Table S1–S5**

**Table S1.** Numbers of bacterial and archaeal species with chemo-organo-heterotrophic (COH), mixotrophic, or non-chemo-organo-heterotrophic metabolism annotated as pathogenic or non-pathogenic.

**Table S2.** Distribution of bacterial phyla across Markov states defined by metabolism and pathogenicity. Isolate counts and within-state proportions are reported for each phylum. Entries labelled “Unassigned” correspond to genomes without a resolved phylum assignment and are shown explicitly. Phyla representing less than 1% of isolates within a state were grouped as “Other”.

**Table S3**. Distribution of bacterial metabolic–pathogenicity Markov states. Values for the clade-pruned bacterial phylogeny are shown in brackets beneath the corresponding values for the full bacterial phylogeny.

**Table S4.** Distribution of archaeal classes across Markov states defined by metabolism and habitat. Genome counts and within-state proportions are reported for each class. Classes representing less than 1% of genomes within a state were grouped as “Other”.

**Table S5.** Counts and proportions of archaeal metabolic–habitat Markov states. Values for the clade-pruned archaeal phylogeny (excluding Halobacteria and Thermococci) are shown in brackets beneath the corresponding values for the full archaeal phylogeny.
