# Supplementary material for: Why are archaea not pathogenic? A hypothesis based on metabolism-habitat covariation
Source: mBio. 2026 Aug 21;17(9):e01185-26. doi: 10.1128/mbio.01185-26 (PMC13556234; doi:10.1128/mbio.01185-26)
Supplement: Supplementary file 2 [file mbio.01185-26-s0009.docx]

**Supplemental materials**

Daniel J. Harrison^a#^, Matthew S. Fullmer^b^, and Nobuto Takeuchi^a,b,c^

^a^School of Biological Sciences, University of Auckland, Auckland, New Zealand

^b^Research Centre for Complex Systems Biology, Universal Biology Institute, University of Tokyo, Meguro-ku, Tokyo, Japan

^c^Department of Biology, Faculty of Sciences, Kyushu University, Fukuoka, Japan

#Address Correspondence to Daniel J. Harrison, dhar522@aucklanduni.ac.nz

**Table S1.** Numbers of bacterial and archaeal species with chemo-organo-heterotrophic (COH), mixotrophic, or non-chemo-organo-heterotrophic metabolism annotated as pathogenic or non-pathogenic.

| **Domain** | **Metabolic annotation^a^** | **Pathogen** | **Non-pathogen** |
| --- | --- | --- | --- |
| Bacteria | Chemo-organo-heterotrophy | 371 | 621 |
|  | Mixotrophic | 11 | 108 |
|  | Non-chemo-organo-heterotrophy | 0 | 138 |
| Archaea | Chemo-organo-heterotrophy | 0 | 463 |
|  | Mixotrophic | 0 | 82 |
|  | Non-chemo-organo-heterotrophy | 0 | 149 |

**a)** Species assigned an unknown metabolic annotation were excluded. For bacteria, this includes 110 pathogenic and 564 non-pathogenic species. For archaea, 112 species were excluded as their growth requirements under experimental conditions are not well characterized, or they remain uncultured.

**Table S2.** Distribution of bacterial phyla across Markov states defined by metabolism and pathogenicity. Isolate counts and within-state proportions are reported for each phylum. Entries labelled “Unassigned” correspond to genomes without a resolved phylum assignment and are shown explicitly. Phyla representing less than 1% of isolates within a state were grouped as “Other”.

| **Markov state** | **Bacterial phylum** | **Isolates (n)** | **Prop. (%)** |
| --- | --- | --- | --- |
| Chemo-organo-heterotroph  & Non-pathogen | Pseudomonadota | 194 | 34.2 |
|  | Bacillota | 112 | 19.7 |
|  | Bacteroidota | 60 | 10.6 |
|  | Actinomycetota | 51 | 9.0 |
|  | Thermodesulfobacteriota | 22 | 3.9 |
|  | Deinococcota | 13 | 2.3 |
|  | Thermotogota | 12 | 2.1 |
|  | Myxococcota | 8 | 1.4 |
|  | Chloroflexota | 7 | 1.2 |
|  | Planctomycetota | 7 | 1.2 |
|  | Spirochaetota | 6 | 1.1 |
|  | Verrucomicrobiota | 6 | 1.1 |
|  | Other (<1%) | 31 | 5.5 |
|  | Unassigned | 39 | 6.9 |
|  |  |  |  |
| Chemo-organo-heterotroph & Pathogen | Pseudomonadota | 359 | 44.6 |
|  | Bacillota | 201 | 25.0 |
|  | Actinomycetota | 61 | 7.6 |
|  | Campylobacterota | 34 | 4.2 |
|  | Spirochaetota | 30 | 3.7 |
|  | Mycoplasmatota | 27 | 3.4 |
|  | Bacteroidota | 25 | 3.1 |
|  | Chlamydiota | 14 | 1.7 |
|  | Other (<1%) | 13 | 1.6 |
|  | Unassigned | 41 | 5.1 |
|  |  |  |  |
| Non-chemo-organo-heterotroph & Non-pathogen | Cyanobacteriota | 46 | 38.7 |
|  | Pseudomonadota | 30 | 25.2 |
|  | Chlorobiota | 8 | 6.7 |
|  | Aquificota | 7 | 5.9 |
|  | Campylobacterota | 7 | 5.9 |
|  | Bacillota | 4 | 3.4 |
|  | Chloroflexota | 3 | 2.5 |
|  | Other (<1%) | 4 | 3.4 |
|  | Unassigned | 10 | 8.4 |

**Table S3**. Distribution of bacterial metabolic–pathogenicity Markov states. Values for the clade-pruned bacterial phylogeny are shown in brackets beneath the corresponding values for the full bacterial phylogeny.

| **Markov state** | **Counts (n)** | **Prop. (%)** |
| --- | --- | --- |
| Chemo-organo-heterotroph & Pathogen | 805 | 54.0 |
|  | (245) | (41.4) |
| Chemo-organo-heterotroph & Non-Pathogen | 568 | 38.1 |
|  | (262) | (44.3) |
| Non-chemo-organo-heterotroph & Non-pathogen | 119 | 8.0 |
|  | (85) | (14.4) |

**Table S4.** Distribution of archaeal classes across Markov states defined by metabolism and habitat. Genome counts and within-state proportions are reported for each class. Classes representing less than 1% of genomes within a state were grouped as “Other”.

| **Markov state** | **Archaeal class** | **Genomes (n)** | **Prop. (%)** |
| --- | --- | --- | --- |
|  |  |  |  |
| COH-capable &  extreme inhabitant | Halobacteria | 266 | 67.3 |
|  | Thermococci | 38 | 9.6 |
|  | Thermoprotei A | 28 | 7.1 |
|  | Methanosarcinia | 24 | 6.1 |
|  | Thermoprotei | 18 | 4.6 |
|  | Thermoplasmata | 7 | 1.8 |
|  | Archaeoglobi | 5 | 1.3 |
|  | Other (< 1%) | 9 | 2.3 |
|  |  |  |  |
| COH-capable &  non-extreme inhabitant | Methanosarcinia | 20 | 57.1 |
|  | Halobacteria | 6 | 17.1 |
|  | Methanomicrobia | 3 | 8.6 |
|  | Methanobacteria | 2 | 5.7 |
|  | Nitrososphaeria | 2 | 5.7 |
|  | Archaeoglobi | 1 | 2.9 |
|  | Methanocellia | 1 | 2.9 |
|  |  |  |  |
| Non-COH & extreme inhabitant | Methanococci | 12 | 26.7 |
|  | Thermoprotei A | 12 | 26.7 |
|  | Methanomicrobia | 8 | 17.8 |
|  | Methanobacteria | 7 | 15.6 |
|  | Nitrososphaeria | 3 | 6.7 |
|  | Archaeoglobi | 1 | 2.2 |
|  | Methanopyri | 1 | 2.2 |
|  | Thermoprotei | 1 | 2.2 |
|  |  |  |  |
| Non-COH &  non-extreme inhabitant | Methanobacteria | 25 | 35.7 |
|  | Methanomicrobia | 18 | 25.7 |
|  | Nitrososphaeria | 15 | 21.4 |
|  | Methanococci | 6 | 8.6 |
|  | Thermoplasmata | 3 | 4.3 |
|  | Methanocellia | 2 | 2.9 |
|  | Methanosarcinia | 1 | 1.4 |

**Table S5.** Counts and proportions of archaeal metabolic–habitat Markov states. Values for the clade-pruned archaeal phylogeny (excluding Halobacteria and Thermococci) are shown in brackets beneath the corresponding values for the full archaeal phylogeny.

| **Markov state** | **Count** | **Prop. (%)** |
| --- | --- | --- |
| COH-capable & extreme inhabitant | 395 | 72.5 |
|  | (91) | (38.7) |
| COH-capable & non-extreme inhabitant | 35 | 6.4 |
|  | (29) | (12.3) |
| Non-COH & extreme inhabitant | 45 | 8.3 |
|  | (45) | (19.1) |
| Non-COH & non-extreme inhabitant | 70 | 12.8 |
|  | (70) | (29.8) |
